# Supplementary material for: Comparative multiomics analysis of cell physiological state after culture in a basket bioreactor
Source: Sci Rep. 2022 Nov 23;12:20161. doi: 10.1038/s41598-022-24687-4 (PMC9686226; doi:10.1038/s41598-022-24687-4)
Supplement: Supplementary file 1 — Supplementary Information 1. [file 41598_2022_24687_MOESM1_ESM.zip › raw data/Transcriptomic raw data/7.AS/2.dasplot/TE1vsTC1/MXE/4_MAPKAPK3_NW_023666041.1_154982218_154982374_-@NW_023666041.1_154981676_154981946_-@NW_023666041.1_154958352_154958491_-@NW_023666041.1_154956965_154957029_-.pdf]

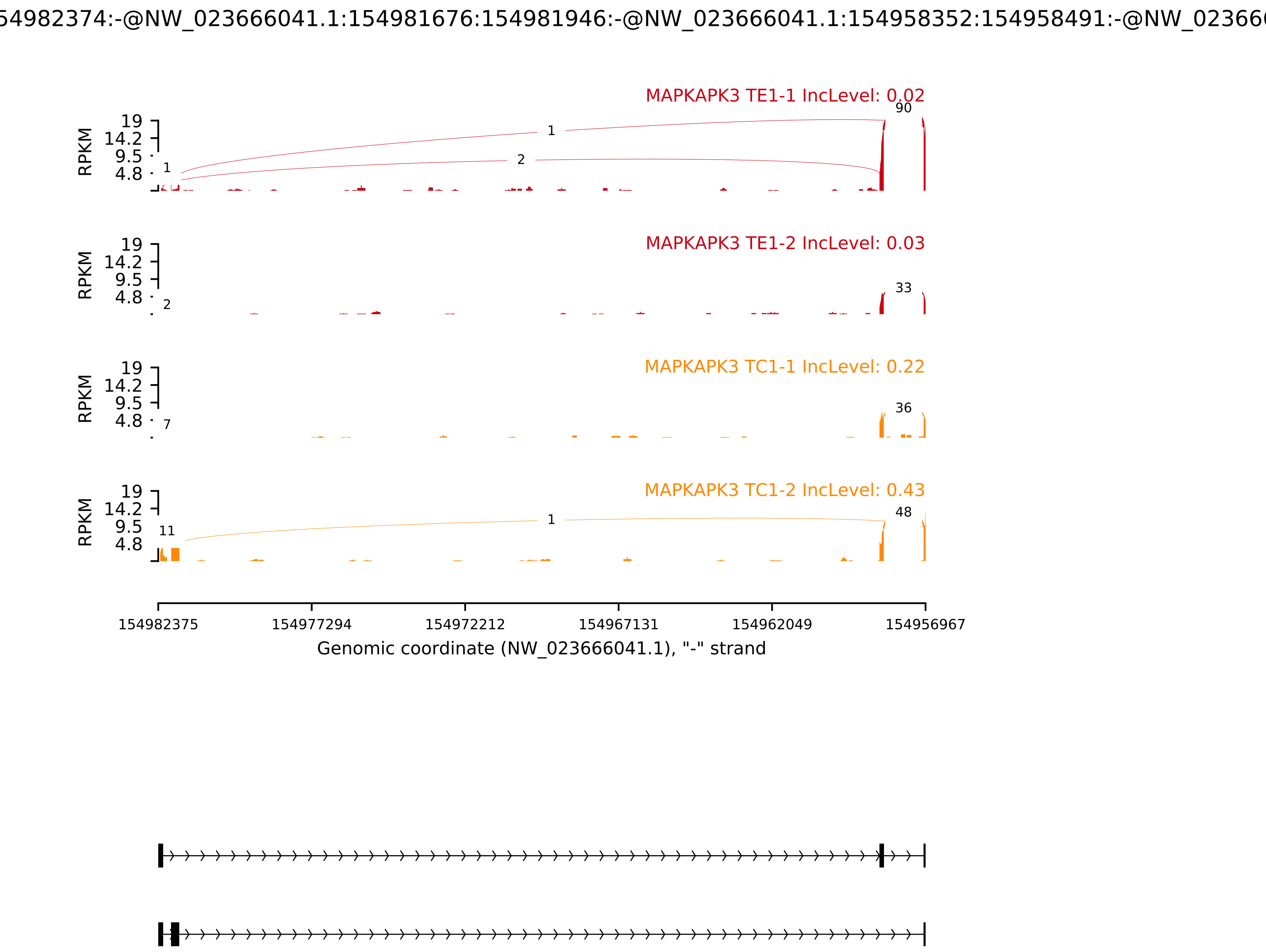
54982374:-@NW_023666041.1:154981676:154981946:-@NW_023666041.1:154958352:154958491:-@NW_02366
MAPKAPK3 TE1-1 IncLevel: 0.02
MAPKAPK3 TE1-2 IncLevel: 0.03
MAPKAPK3 TC1-1 IncLevel: 0.22
MAPKAPK3 TC1-2 IncLevel: 0.43
RPKM
19
14.2
9.5
4.8
154982375
154977294
154972212
154967131
154962049
154956967
Genomic coordinate (NW_023666041.1), "-" strand
